# Feasibility of Consumer Grade GNSS Receivers for the Integration in Multi-Sensor-Systems

**DOI:** 10.3390/s20092463

**Published:** 2020-04-26

**Authors:** Tobias Kersten, Jens-André Paffenholz

**Affiliations:** 1 Institut für Erdmessung, Leibniz University Hannover, 30167 Hannover, Germany; 2 Institute of Geotechnical Engineering and Mine Surveying, Clausthal University of Technology, 38678 Clausthal-Zellerfeld, Germany; jens-andre.paffenholz@tu-clausthal.de

**Keywords:** GNSS, high sensitivity GNSS receiver, multi-sensor-system, direct geo-referencing, laser scanning

## Abstract

Various GNSS applications require low-cost, small-scale, lightweight and power-saving GNSS devices and require high precision in terms of low noise for carrier phase and code observations. Applications vary from navigation approaches to positioning in geo-monitoring units up to integration in multi-sensor-systems. For highest precision, only GNSS receivers are suitable that provide access to raw data such as carrier phase, code ranges, Doppler and signal strength. A system integration is only possible if the overall noise level is known and quantified at the level of the original observations. A benchmark analysis based on a zero baseline is proposed to quantify the stochastic properties. The performance of the consumer grade GNSS receiver is determined and evaluated against geodetic GNSS receivers to better understand the utilization of consumer grade receivers. Results indicate high similarity to the geodetic receiver, even though technical limitations are present. Various stochastic techniques report normally distributed carrier-phase noise of 2 mm and code-range noise of 0.5–0.8 m. This is confirmed by studying the modified Allan standard deviation and code-minus-carrier combinations. Derived parameters serve as important indicators for the integration of GNSS receivers into multi-sensor-systems.

## 1. Introduction

**Figure 1:**
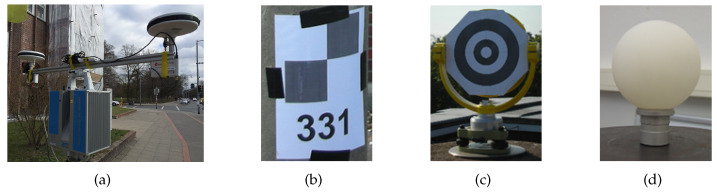
Multi-sensor system (MSS) for direct geo-referencing and different kinds of marker types for control point transformations of 3D point clouds; (**a**) Zoller+Fröhlich laser scanner with consumer market GNSS equipment, (**b**–**d**) several realisations for control point markers.

Instantaneous three-dimensional (3D) point clouds recorded with stationary, terrestrial laser scanners (TLS) generally refer to a local horizon system in which the position and orientation relative to a super-ordinate coordinate system is transferred to the necessary geodetic datum only by pre-calculated control points (cf. Figure 1b–d). A multi-sensor system (MSS) to enable direct geo-referencing is designed by [1]. The current MSS configuration consists of cost-effective and weight-reduced elements such as two ublox NEO-M8T receivers combined with Ashtec L1 antennas. The antennas are mounted on the laser scanner with a baseline of approx. 1 m, optimized to the present field of view (cf. Figure 1a). An Extended Kalman Filter (EKF) approach with an incorporated recursive Kalman filter is implemented to transform the parameters from the local to the global reference frame.

This configuration only leads to optimal results if the system noise is quantified and precisely known at the level of the original observations. Hence, the consumer units are characterised, functional and stochastic models can be used to improve the modelling of observables that result in optimal position solution in an update of the current MSS set-up. Thus, the following prerequisites are required and identified: (1) normal distributed observables, (2) information for the co-variance matrix and (3) no correlations in time should be present. They are not completely fulfilled in the current ensemble, as stochastic dependencies by temporal correlations in the state vector of the EKF are present. In addition, previous studies elaborated that the kinematic time series of a 360${}^{{}^{\circ}}$ rotation of the laser scanner has additional dependencies in the context of auto-correlation studies. Thus, a comprehensive benchmark analysis is needed to quantify the system properties of the high-sensitivity receivers and to derive verifiable statements for the parameters of the EKF-modelling. In addition, this benchmark study provides robust values for noise figure and noise behaviour of consumer grade GNSS receivers of kind ublox NEO-M8T with respect to the geodetic counterparts.

As over the last decade the development of consumer market and embedded GNSS systems increased significantly, the advantages like, for example, small-scale, low-power consumption and cost efficiency, are raising more interest in several communities, mainly focusing on geomatics and navigation. A number of publications have been published, dealing with the general feasibility and usability of consumer GNSS receivers for geomatics engineering approaches like, for example, Benoit et al. [2], Schwieger [3], Weston and Schwieger [4], Håkansson [5], Hohensinn and Geiger [6], Paziewski et al. [7]. Studies of Benoit et al. [2] from a small scaled network with up to ten receivers reported precisions of 3 mm for the horizontal and up to 5 mm for the vertical components using the GNSS carrier phases. Similar results and studies are available by Cina and Piras [8], Biagi et al. [9], Poluzzi et al. [10]. In addition, Krietemeyer et al. [11], Douša et al. [12] examine the potential of wide-spread deployment of consumer grade GNSS receivers in data sparse regions with limited investment budgets.  Wilkinson et al. [13] demonstrate consumer grade GNSS equipment as an accurate tool in short baseline networks (below 10 km) as they successfully resolve near-field co-seismic displacements. Economic implementations beyond solely scientific approaches are implemented among others in Schröder [14], Stempfhuber and Alberding [15]. However, a comprehensive characterization of consumer GNSS receivers relative to their geodetic counterparts is at present not available in the literature.

All reported approaches have one idea in common: finding the right balance between the advantages of high-end and consumer market GNSS equipment. These aspects are described by their properties, like, for example, individual characteristics, their noise floor with respect to the original observations and the parameters of the noise.

Addressing those parameters and answering these open questions is the objective of this paper. We present and discuss a concept for a benchmark study of consumer grade GNSS receivers with focus on our individual MSS ensemble. In contrast to the majority of papers that deal with the feasibility of consumer grade GNSS equipment in geomatics, engineering and navigation applications, our study addresses both the observation and the position domain in order to achieve representative results. Findings obtained by our approach will assist future implementations to optimally quantify and tune important system parameters not only for MSS but to furthermore reliably obtain properties of high-sensitivity receivers.

The concept of the paper is as follows. The next section presents the laboratory set-up, with focus on details of the data recording as well as the required data pre-processing. The third section will discuss the studies on the observation domain whereby the fourth section discusses the results on the position domain (i.e., geodetic parameters). The fifth section summarises the findings, and the sixth section closes the paper.

## 2. Consumer GNSS Receiver Benchmark Study

### 2.1. Setup and Basis of Analysis

Because there are currently no common terms used consistently for a class of low-cost or cost-efficient devices, we define in this paper the term *consumer GNSS receiver* when referring to the properties, characteristics and capabilities of the analysed NEO-M8T receivers.

**Figure 2:**
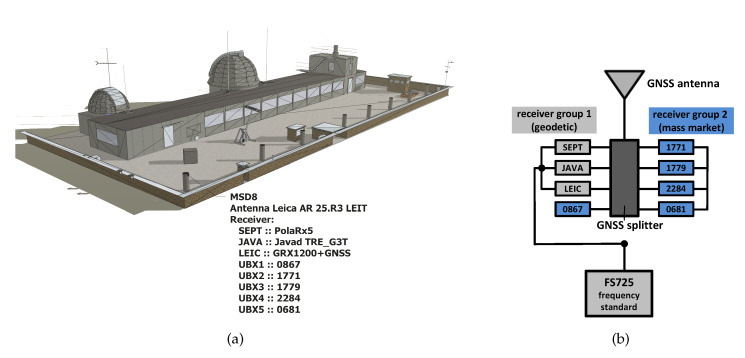
Zero baseline set-up to study the equipment-specific characteristics of consumer market GNSS receivers; laboratory network (**a**) and configuration of receiver groups (**b**).

The benchmark analysis with both geodetic and consumer GNSS receivers are obtained at the laboratory GNSS network of the Institut für Erdmessung (IfE), Leibniz University Hannover (LUH, cf. Figure 2a). There, a permanent reference station (MSD8) equipped with a broadband Leica AR25.R3 3d choke ring antenna, ready to receive signals of different GNSS, is operated by IfE. The antenna is connected to an eight-way splitter with eight GNSS receivers connected to its outputs (cf. Figure 2b). Thus, all receivers are connected to the same antenna and receive the common GNSS signal. This zero baseline set-up warrants to access the individual properties and characteristics of the receivers. In addition, the geodetic receivers are connected to an external rubidium frequency standard (FS725). The configuration of the benchmark set-up is depicted in Figure 2b. Figure 2b and shows that two groups of different kinds of GNSS receivers are used to evaluate the characteristics of several combinations and cross-combinations between three geodetic GNSS receivers (Septentrio PolaRx5TR, Javad Delta TRE_G3T, Leica GRX1200+GNSS) and five consumer grade GNSS receivers (ublox NEO-M8T) and among each other. To keep consistency, consumer receivers are assembled to a geodetic grade antenna although they are not designed for such combination. The main difference between consumer antennas and geodetic antennas are the overall antenna assembly, design, gain, phase pattern stability and mitigation of station dependent effects and reflections [16,17]. However, to ensure the correct current at the low noise amplifier (LNA) of the geodetic antenna, a geodetic receiver feeds the antenna with 100 mA as required by antenna specifications.

The most interesting characteristic of such a class of consumer grade GNSS receivers is shown by their high sensitivity, which means their capability to track very weak signals with respect to geodetic receivers. Several studies at IfE like, for example, Bochkati and Schön [18], prove that those class of receivers track significantly more satellites relative to geodetic receivers. These findings were evaluated under urban conditions (urban canyon) analysing only the GPS C/A L1 signal. However, at first sight, the quality is questionable. However, using moderately adapted data pre-processing steps, more usable satellites are available at second sight.

Table 1 summarises the GNSS receiver test samples of the benchmark study. For reasons of comparability, we add the type of firmware, timing module and measurement engine, too. The firmware of the timing modules for NEO-M8T receivers changes from TIM 1.00 to TIM 1.01 in the year 2017, reducing inter-frequency biases on the carrier phase, which have been present for some time. All receivers in Table 1 support the antenna’s low noise amplifier (LNA) with 100 mA.

Consumer grade GNSS receivers are configured for static data recording and use a carrier-to-noise ratio (C/N0) threshold of 5 dB-Hz (factory settings). Geodetic receivers are configured by their individual factory default settings. The most interesting properties are the bandwidth of the tracking loops, which have not changed with respect to the factory settings. Thus, the Javad Delta receiver utilizes bandwidth of 25 MHz and the Septentrio 10 MHz. Parameters of the bandwidth for the Leica receiver are neither reported and published nor accessible. We suggest a bandwidth of tracking loops of 10 MHz.

The set-up in Figure 2b allows statements for several studies: On the one hand, individual properties for a mixture of consumer and geodetic receivers are accessible. On the other hand, the performance of consumer GNSS receivers among each other can be elaborated, to access the relative performance parameters and observation noise. An external frequency standard for the geodetic GNSS receivers ensures the separation of observation noise for the comparison relative to consumer GNSS receivers.

GNSS data is recorded for seven 24-hour sets between DOY67 and DOY73 in 2018. Considering and following the RINEX 3 [19] conventions, the following observation types on L1, C/A signal are recorded and used: *code ranges:* GC1C, RC1C, *carrier phases:* GL1C, RL1C, *Doppler:* GD1C, GL1C and *C/N0:* GS1C, RS1C.

### 2.2. Pre-Processing of and Accessibility to the GNSS Dataset

To pre-process the observations, collected in proprietary binary container formats, the RINEX 3.0x format is used [19]. To convert the ublox binaries, the convbin tool of the RTKLib software (version 2.4.2 R13) [20] is applied. The used RTKlib version is able to access the RAWX (multi-GNSS) observation container format of the ublox binary data files.

Consistency checks are performed using the RINEX tool gfzrnx [21] provided by the Helmholtz Centre Potsdam German Research Centre for Geosciences (GFZ Potsdam). Further data analysis and processing is studied using the IfE-GNSS-Toolbox (version 6.1). The IfE-GNSS-Toolbox is maintained and implemented by the working group Positioning and Navigation group (Prof. Schön) at the IfE, LUH.

The data of the benchmark study are published [22] as open access in the LUH institutional repository. The authors encourage the community to work with the dataset, which is available free of charge and provides all required meta-data and additional information.

## 3. Observation Domain

### 3.1. The Role of Carrier-to-Noise Ratio As Quality Indicator for GNSS Receivers

**Figure 3:**
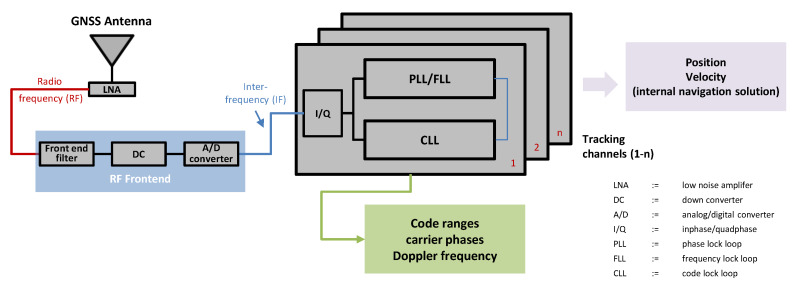
GNSS signal processing chain inside a receiver from the antenna to the tracking loops.

The C/N0 is an overall quality indicator of a GNSS receiver and its tracking performance. Therefore, we will have at first a close look on the insights of a GNSS receiver (cf. Figure 3). The pseudo-range between a satellite and a receiver is derived by correlating the pseudo-random noise (PRN) code from the satellite with the PRN code sequence, provided internally in the receiver by tracking loops. However, the process of acquisition and tracking of a single satellite and signal is a two dimensional code-carrier-signal replication process [23], as the carrier frequency plus Doppler have to be correlated in a carrier phase loop, which can be implemented as a phase locked loop (PLL) or a frequency locked look (FLL).

Each of the two tracking loops from Figure 3 are connected to and complements the other: While the code lock loop (CLL) is used to synchronize the received signal, the PLL/FLL synchronizes the frequency of the signal. Frequency synchronization is particularly important during down converting (DC), as the receiving signal may be Doppler shifted by a movement of the receiver. The implemented discriminator in the PLL/FLL adjusts this movement. In the case of static receivers this movement is caused by the satellites only.

The tracking loops require a specific signal strength with respect to receiver noise and interfering signals. This quality—or capacity of the receiver—is indicated by the C/N0 density ratio. The C/N0 is a normalized measure of signal-to-noise ratio and a clear indicator of carrier phase and code range precision. The minimum C/N0 indicates the threshold up to which the PLLs/FLLs are still able to track the received GNSS signals. Below this certain C/N0 threshold, the receiver loses the lock to the signals, which makes the tracking impossible [24]. Generally, the PLL/FLL stops working as the phase error increases by the increasing of both, phase jitter and dynamic stress.

The lowest C/N0 threshold is not a fixed value but dependent on the adjustment of the bandwidth of the tracking loops. Narrowing the bandwidth of the tracking loops, on the one hand, results in better anti jamming but also in sluggish response time (applicable for static scenarios). Expanding the bandwidth, on the other hand, results in more accurate responsiveness to high accelerations (applicable in kinematic scenarios) but will increase phase jitter [23,24]. The parameters incorporating the performance of the phase jitter for a PLL are described in [24] by the standard deviation $\sigma_{t_{PLL}}$ as

(1) $$\sigma_{t_{PLL}}=\frac{360^{{}^{\circ}}}{2\pi }\sqrt{\frac{B_{n}}{C/N0}\left[1+\frac{1}{2T\frac{C}{N0}}\right]}$$

with the carrier loop bandwidth $B_{n}$ in Hz, C/N0 in $10^{\frac{C/N0}{10}}$ expressed in decibel Hertz (dB-Hz) and *T* the prediction integration time in seconds. From Equation (1), the dependencies between the C/N0 are deduced. By the given bandwidth $B_{n}$ and integration time, the C/N0 influences the phase jitter and defines the threshold of the ability to track and acquire satellite signals. Therefore, the C/N0 points out to be an important property of the overall performing of GNSS receivers and have be considered carefully.

Kaplan [24] derives the threshold for GPS L1 at C/A signal by the typical Costas loop and gives a threshold for GPS receivers of 25 dB-Hz for $B_{n}$ of 20 MHz and an *T* of 20 msec. These values are quite comparable with those from the used receiver, as shown in Table 1 taking into account individual variations of the $B_{n}$ and *T* parameters (cf. Section 2.1). However, the C/N0 is dependent, in addition, on several sources, for example, components in the front-end, the low noise amplifier (LNA), filter implementations in the tracking loops, losses in cables and the antenna gain.

**Figure 4:**
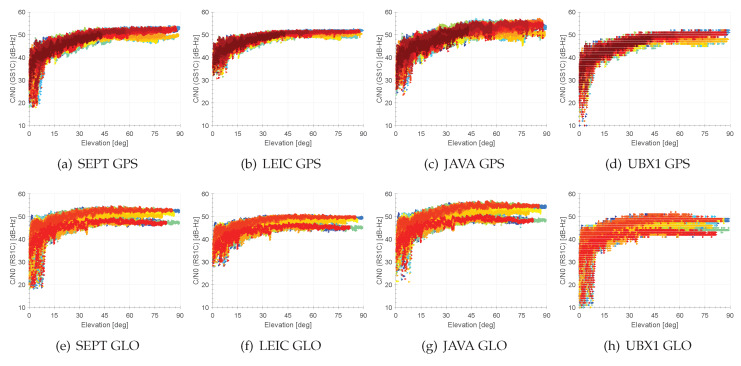
Signal characteristics of the analysed receivers for GPS (**a**–**d**) and GLONASS (**e**–**h**) of frequency L1 on the C/A signal.

Results of individual C/N0s versus the elevation for geodetic and consumer GNSS receivers are summarised in Figure 4 and Table 2. Although the NEO-M8T receivers only provide a subdivision of C/N0-values of 1 dB-Hz with respect to geodetic receivers that provide 0.1–0.25 dB-Hz, the overall performance is detectable. Individual C/N0 thresholds of geodetic GNSS receivers indicate a sharp cut-off at individual values between 20–30 dB-Hz. Consumer GNSS receivers show a very low threshold of 5 dB-Hz for elevations below 10${}^{{}^{\circ}}$. Expect for the LEICA receiver (cf. Figure 4b) all other receivers provide the same deviation of C/N0 values at elevations above 15${}^{{}^{\circ}}$. Biases in C/N0 curves of 2.5–5 dB-Hz are present and relate to the individual GPS satellite generations, as the signal strength varies slightly. Similar conclusions and findings are shown for GLONASS C/N0s (cf. Figure 4e–h)). Table 2 shows, that the deviations at specific elevation bins are closely comparable between the geodetic and consumer grade GNSS receivers and show magnitudes of 5 dB-Hz at low elevations and 3–6 dB-Hz at high elevations.

In summary, the overall performance of the consumer GNSS receiver is well comparable to their geodetic counterparts. The prominent difference is the capability of the consumer grade GNSS receivers to track signals with a very low C/N0. Hence, they are sometimes known as high sensitivity receivers. However, the C/N0s are an important indicator for the overall signal quality of code ranges and carrier phases. Therefore, the consideration of C/N0s for weighting GNSS observables as described below and by others in Wieser and Brunner [25], Kersten and Schön [26], are very advantageous. Necessarily, C/N0 reference curves are required for the combination of antenna and receiver (possibly with cable), which have to be determined beforehand.

### 3.2. Double Differences for Code Ranges and Carrier Phases

Additionally, double difference (DD) residuals for both code range and carrier phase residuals are an important quality indicator for the comparison of geodetic and consumer grade GNSS receivers.

**Figure 5:**
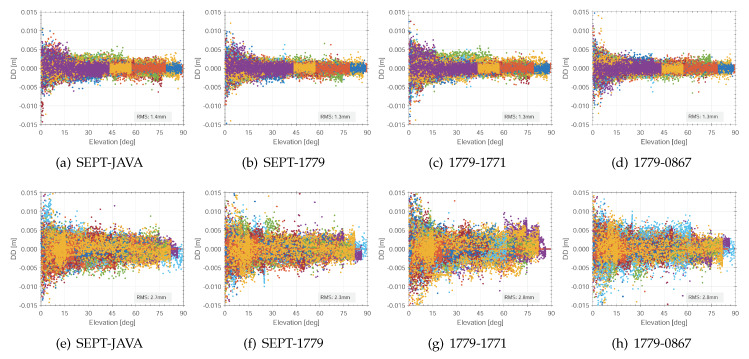
Double difference residuals of the carrier phase L1 C/A versus the elevation for selected receiver combinations on the frequency L1 and the signal C/A, system GPS (**a–d**), GLONASS (**e–h**).

In a first step, we will start analysing the carrier phase residuals of GPS and GLONASS. Therefore, different combinations of the carrier phase residuals are analysed; see the results in Figure 5. The residuals for the combination of SEPT-JAVA gives a first rough impression of the comparability of geodetic GNSS receivers. Different manufactures follow different strategies concerning the implementation of GNSS signal tracking. Here, residuals follow a normal probability density function. Maximum but few values of DD carrier phase residuals are around 5 mm whereas the residuals value for the DD residuals for 24 hours are given by 1.4 mm (cf. Figure 5a).

Interestingly, the combination of geodetic and consumer GNSS receivers do not change significantly for GPS, as indicated by Figure 5b. Here, we detect some more outliers also in higher elevations, but the overall residuals are very well comparable to the zero baseline of geodetic receivers. This indicates that the observation quality versus geodetic receivers do not differ much. Prominent differences occur for the elevation range below 15${}^{{}^{\circ}}$ cf. Figure 5a,b. Furthermore, the combination of two consumer grade GNSS receivers provide similar carrier phase residuals with values of 1.3 mm, too.

**Figure 6:**
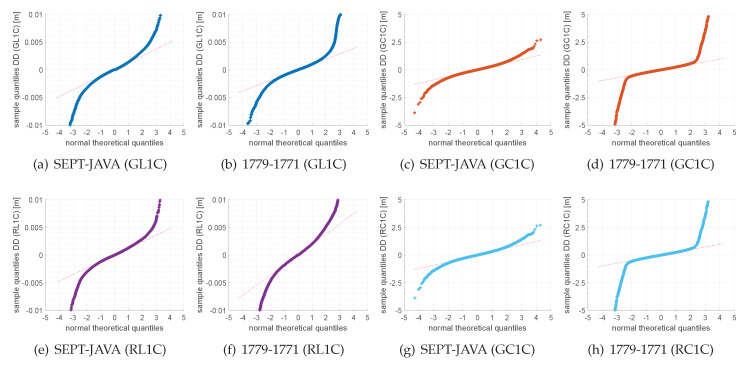
Quantile–quantile plots for the analysis of normal distribution of observed-minus-computed double difference residuals on a short baseline between geodetic receivers and consumer grade receivers for systems GPS and GLONASS.

In comparison to GPS, the residuals of GLONASS are slightly higher than for GPS with maximum values of up to 7–10 mm. In cases of the combination of consumer grade receivers only, the GLONASS residuals reach values of up to 7 mm, although individual GLONASS frequencies are consistently corrected in the IfE-GNSS-toolbox. However, the DD residuals follow a normal distribution with residuals of 2.7–2.9 mm, also verified by the quantile–quantile (qq) plots in Figure 6e–f.

**Figure 7:**
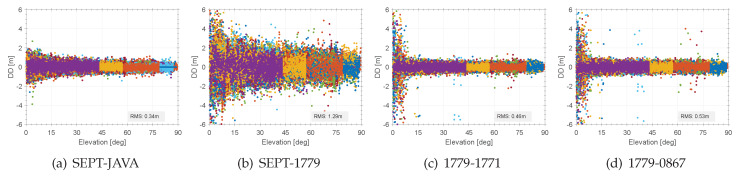
Double difference residuals of code observables on C/A versus the elevation for selected receiver combinations on the frequency L1 and signal C/A, system GPS.

Even more interesting in this context is the different behaviour of the code range DDs that are shown in Figure 7. Here, the most important impact of combining or not combining geodetic and consumer GNSS receivers is prominent and obvious. The reference are the code range DDs of geodetic receivers, which results into low number of outliers and residuals of 0.3 m, which fits pretty well to the expectable code noise on the C/A signal (cf. Figure 7a). The elevation dependency and higher noise at low elevation bins is meaningful as different receiver types and different signal tracking techniques are compared.

The mixed combination results to residuals of code range DDs with magnitudes of up to 2.5 m, which is four times higher than for the geodetic receivers (cf. Figure 7b). However, using consistent equipment and signal tracking techniques, the residuals for the consumer GNSS receiver result into values of 0.4–0.5 m (1.6 times higher than for the geodetic receivers), as shown by Figure 7c–d. A small number of outliers are detectable. Important to notice is, that among all combinations, the consumer GNSS receivers show a prominent noise below an elevation of 8${}^{{}^{\circ}}$–10${}^{{}^{\circ}}$. Hence, this prominent noise is closely related to the low C/N0 threshold value within the analysed NEO-M8T receivers that are present at Figure 4d,h at the same elevation bins. Nevertheless, there are no systematic deviations detectable in the code range DDs as the studies consumer receivers are from the same kind and use the identical signal tracking technique.

### 3.3. Code-Minus-Carrier (CMC)

From previous sections, it was figured out that the consumer grade receiver can achieve the same level of precision relative to carrier phase noise and code noise as the geodetic grade receivers but they mainly seem to depend on the chosen receiver combination. In addition, the implementation and design of the tracking loops for both, carrier phases and code ranges introduce a significant impact on the noise and quality of the obtained data. To further study the resulting code noise for the different GNSS receiver combinations, we analyse the CMC combinations in more detail [27,28].

The CMC from a ground station *A* and a satellite *j* can be expressed as

(2) $$\begin{array}{ccc} CMC_{A}^{j} & = & P_{A}^{j}-\lambda \varphi_{A}^{j}\\ & = & -\lambda N_{A}^{j}-2I_{A}^{j}+\Delta d_{A_{P,\varphi }}+\Delta d_{P,\varphi }^{j}+MP_{A,P}^{j}-MP_{A,\varphi }^{j}+\epsilon_{P}-\epsilon_{\varphi } \end{array}$$

with the wavelength λ, the carrier phase observation φ, the code-range *P*, the carrier phase ambiguities $\lambda N_{A}^{j}$, the double of the ionospheric effect $2I_{A}^{j}$, the delays at each station (satellite and ground) for both, carrier phase and code range ($\Delta d_{A_{P,\varphi }},\;\Delta d_{P,\varphi }^{j}$), the multipath for code $MP_{A,P}^{j}$ and carrier phases $MP_{A,\varphi }^{j}$ as well as their corresponding noise $\epsilon_{\varphi }$ and $\epsilon_{\varphi }$. These observations contain numerous systematic error components that initially deny any access to the information of interest. As we are interested in a differential solution and would like to analyse the differential behaviour of the receiver combinations, it is justified to form receiver-to-receiver single differences of the CMC between two stations *A* and *B* as

(3) $$\begin{array}{ccc} \Delta CMC_{A}^{B} & = & CMC_{A}^{j}-CMC_{B}^{j}\\ & = & \Delta MP_{{A,B}_{P}}^{j}+\Delta MP_{{A,B}_{\varphi }}^{j}+\Delta d_{{A,B}_{P}}^{j}+\Delta d_{{A,B}_{P,\varphi }}+\epsilon_{{A,B}_{P}}+\epsilon_{{A,B}_{\varphi }}. \end{array}$$

Thus, the error components reduce considerably. The observation $\Delta CMC_{A}^{B}$ now contains the differential hardware delays of the receivers ($\Delta d_{{A,B}_{P}},\;\Delta d_{{A,B}_{\varphi }}$) as well as the differential multipath components $\Delta MP_{{A,B}_{P}}^{j},\;\Delta MP_{{A,B}_{\varphi }}^{j}$ caused by different signal tracking approaches and the non-modelled noise. The noise is superimposed by the code-range noise $\epsilon_{{A,B}_{P}}$, which makes this observation suitable for further studies.

**Figure 8:**
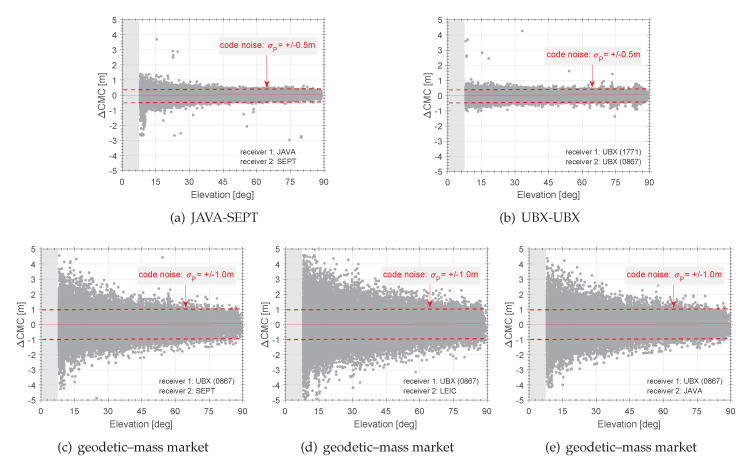
Differential CMC (ΔCMC) residuals for several combinations of consumer grade and geodetic receivers versus elevation. An elevation mask of 8${}^{{}^{\circ}}$ is applied.

**Figure 9:**
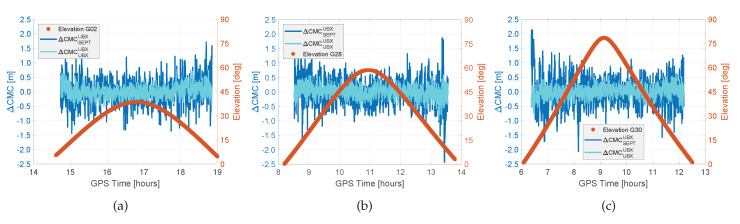
Differential CMC (ΔCMC) residuals for both, consumer grade and geodetic receiver combinations for satellites of different elevation on DOY067, 2018; (**a**) low elevation, (**b**) mean elevation, (**c**) high elevation. Initial offsets are eliminated for each pair of receivers.

The complete dataset, which contains seven days [22], provides several possible combinations, which we studied in detail. During the analysis, we figured out, that the behaviour of the certain combinations of $\Delta CMC_{A}^{B}$ is very well described and repeated for each receiver combination on consecutive days. Therefore, we summarise the results in Figure 8 and Figure 9, exemplary for some combinations and satellites in DOY067, 2018.

In Figure 9, the differences in the expected noise of the code observations can be achieved. The ΔCMC show zero mean with repeatable noise magnitudes of approx. 1 m for the combination of geodetic and consumer receiver combination $\Delta {\mathrm{CMC}}_{SEPT}^{UBX}$. The combination of the same kind of receiver like in cases of $\Delta {\mathrm{CMC}}_{SEPT}^{UBX}$ show a code noise of less than 0.5 m. These findings are independent of the chosen satellite (cf. Figure 9) and are repeatable for all combinations and recorded days.

The consistency of ΔCMC versus all satellites is validated in Figure 8 for different receiver combinations versus elevation. Here, an elevation mask of 8${}^{{}^{\circ}}$ is commonly applied to all combinations. For reasons of comparison, Figure 8a displays the expectable observation noise for geodetic receivers whereby Figure 8b demonstrates the noise for consumer grade receivers. The resulting elevation dependency is meaningful as different receiver manufactures implement different tracking algorithms, which introduce systematic differences between the obtained receivers (cf. Figure 8a). In contrast, there is no elevation dependency for the consumer grade receivers, as in this case, receivers of the same kind and tracking algorithm approach are differentiated (cf. Figure 8b). This result is the benchmark for the comparison of the consumer market and geodetic receivers. Hence, Figure 8c–e indicate, that the code range noise raises up to ±1 m. These results show the differential impact of the receiver’s signal tracking characteristics that are challenging when combining these different kind of receiver types. Different signal tracking and propagation of multipath signals as well as ionospheric effects mainly superimpose the impact. However, the noise significantly reduces when the same kind of receiver is in use. Thus, this indicates that using consistent equipment is much more important when applying and configuring differential GNSS applications with consumer grade receivers.

### 3.4. Allan Standard Deviation

The noise characteristics of analysed receivers (DD time series) are studied, using the Allan variance $\sigma_{y}^{2}\left(\tau \right)$ [29,30], which is defined as the infinite time average of squared differences of consecutive fractional values from a time series ${\bar{y}}^{\tau }$, averaged over a certain time interval τ with

(4) $$\sigma_{y}^{2}\left(\tau \right)=\frac{1}{2}\langle {\left({\bar{y}}^{\tau }\right)}^{2}\rangle$$

where $\langle \ldots \rangle$ defines the infinite time interval. The Allan standard deviation $\sigma_{y}\left(\tau \right)$ is simply the square root of the Allan variance. In general, the Allan variance is widely used as a key tool to analyse non-stationary effects in frequency time series to characterise atomic clocks, oscillators and frequency stability in the time domain [30]. The concept for the analysis of noise properties in successive time series of observations, however, remains the same and is adopted here. The Allan variance is advantageous to study higher order differences, as they are mostly stationary while the original process is not. Hence, the concept of the structure function of the second differences is comparable to the Allan standard deviation [31] and principles and conclusions from the power spectral density are closely related to the properties of the Allan standard deviation [30]. In this benchmark test, we study the modified Allan standard deviation $\mathrm{mod}\;\sigma_{y}\left(\tau \right)$, which distinguishes between white phase noise and flicker phase noise to strictly separate these noise characteristics.

**Figure 10:**
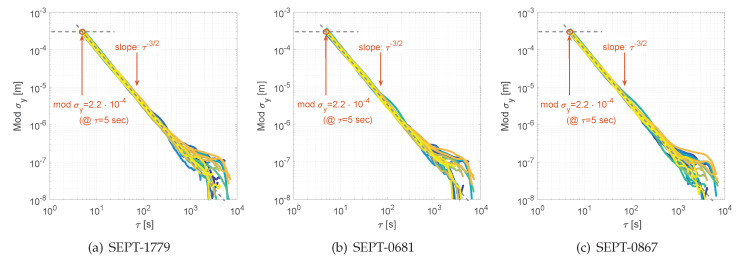
Modified Allan Standard Deviation shown for different GNSS receiver combinations to analyse the stochastic characteristics of the DD carrier phase observables form a zero baseline for the signal GL1C (GPS) on DOY067, 2018.

Comprehensive data cleaning, data checks and removal of all data-gaps results in a valid input to process for the modified Allan standard deviation. These findings are summarised in Figure 10 for GPS carrier phase DD residuals on C/A signal and for several mixed combinations of GNSS receiver types.

The modified Allan standard deviation supports our statements and findings we obtained by the DD time series, the normal distribution and the qq-plots (cf. Section 3.2). As the data sets are processed for all seven consecutive days, the findings are repeatable and similar and are shown exemplary by Figure 10 for DOY067, 2018. For all combinations, we figured out a clear white noise process for the carrier phase time on the GPS C/A signal with a slope of $\tau^{-3/2}$ [30]. Individual offsets are induced by the change of reference satellites, which introduce slightly different noise, as the time spans of each reference satellite is not equal below each other. Nevertheless, we obtained a minimum value of $\sigma_{y}\left(\tau \right)=$ 1.25 mm and a maximum value of $\sigma_{y}\left(\tau \right)=$ 3.5 mm at τ= 1 s sampling interval for the mixed combination SEPT-NEO-M8T. This results in a mean value of 2.35 mm for solely white noise on carrier phase DD residual time series.

Furthermore, the findings confirm our former studies on consumer grade GNSS receivers [32]. There, on a zero baseline, a white noise process for GPS L1 on C/A was obtained to $\sigma_{y}\left(\tau \right)=$ 4 mm at τ=1 s. In this study, different reference receiver and different firmware for the NEO-M8T receiver have been used. In addition, the DD carrier phase residual time series were recorded using an interval of 5 s.

### 3.5. GNSS Signal Splitter and Its Characterization

**Figure 11:**
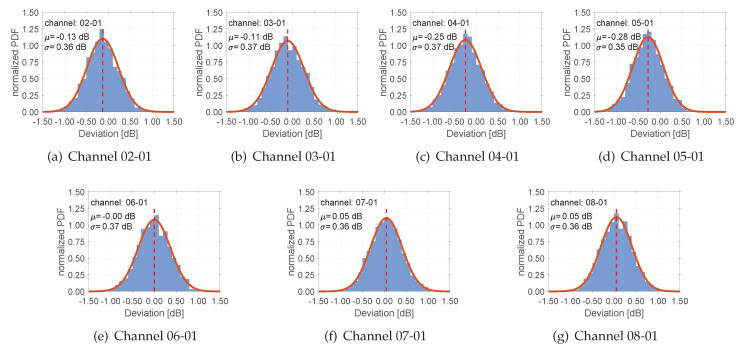
Deviations obtained for the GNSS-signal splitter by laboratory measurements; individual channel variations are summarised w.r.t. their normalised normal probability density function (PDF). The PDFs are referenced to the first channel 01 (DC true) and shown in sufigures (**a**–**g**) for each combinations in relation to the other inputs of the eight-way splitter (DC false). The impact of the mean μ value and the standard deviation σ are below the level of significance.

In addition to the benchmark studies, we analysed the instrumental impact of the GNSS signal splitter, too. Therefore, we checked the delays and the differential signal strength at the individual outputs of the active eight-way splitter from the company GPS networking. The obtained results are summarised in Figure 11, where the variations between the individual outputs with respect to the first channel are shown. Here, the first channel serves as power supply to support the active splitter, which is connected with the SEPT receiver (cf. Figure 2b) during our benchmark study. The distributions of the differences at individual splitter outputs coincide to a normal distribution probability. The individual mean values μ and standard deviations σ are presented in addition. The maximum mean value results in μ= 0.28 dB, the minimal value coincides perfectly with μ= 0.0 dB. The standard deviations are comparable for all the differential time series and outputs and are between σ= 0.35–0.37 dB. In conclusion, we conclude that the splitter used in the benchmark study does not create any additional uncertainties.

## 4. Position Domain

In addition to comprehensive analysis with respect to the observables, a study on the position domain is elaborated to prove and cross-check the performance of individual receivers and their mixed combinations on a zero baseline. Therefore, we used the Bernese 5.2 GNSS software [33] and orbits [34] from by the Centre of Orbit Determination in Europe (CODE). The ambiguities are fixed to 100 percent using the SIGMA-Method [33], and elevation dependent weighting of observables is used in static mode.

Data of all seven days from the GNSS benchmark study [22] is used to analyse the derivations in the position domain. A combined GPS/GLONASS L1 solution is processed and the final solution was obtained using ADDNEQ2 with the mean of the daily stacked normal equations.

**Figure 12:**
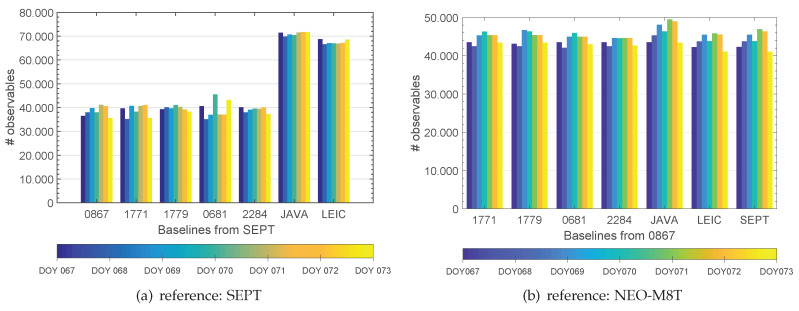
The maximum number of available observations for DD calculation in Bernese GNSS software depends on the number of available tracking channels thus resulting in dependencies in the combination of receivers, (**a**) SEPT as reference, (**b**) NEO-M8T receiver (0867) as reference.

Figure 12 and Table 1 indicate different numbers of tracking channels among the studied GNSS receivers. Thus, differences in the maximum values of available observations in the Bernese processing differ significantly (cf. Figure 12a versus Figure 12b). Results are obtained by only changing the reference station from SEPT (Septentrio PolaRx5TR) to 0867 (NEO-M8T). Individual differences of daily data recording under identical conditions are noticeable and show differences of around 1000–2000 observables (cf. Figure 12b).

Around 40,000 observables are available for the combination of consumer grade and geodetic grade receivers (SEPT-UBX, cf. Figure 12b). On the contrary, for the combination of two geodetic grade receivers, this magnitude is at around 70,000 (cf. Figure 12a). That indicates differences on the position domain. The impact of the reference receiver and the performance of the baseline solutions relative to the used receivers are therefore elaborated for two sets: on the one hand with the reference receiver (A) as consumer GNSS receiver NEO-M8T (0867) and on the other hand with a geodetic receiver Septentrio PolaRx5TR GNSS receiver. The baseline solution (B) serves as reference for this comparison.

**Figure 13:**
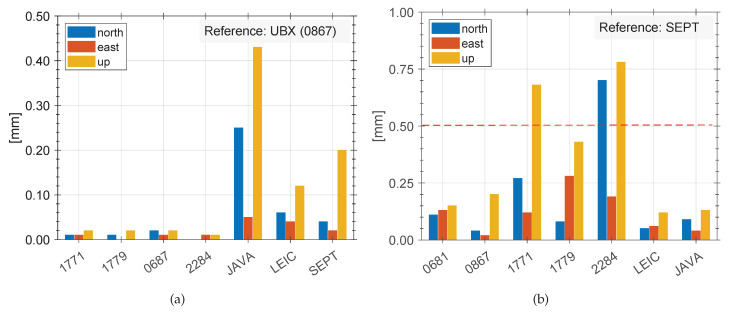
Position solution obtained from seven daily batches on a short baseline, with reference SEPT (**a**) and NEO-M8T 0867 (**b**). The dashed line in Figure 13a indicates half of the level of repeatability of Figure 13b.

In both cases, the ambiguities are perfectly fixed to 100%. Differences between the final coordinate solution and the reference are present. Figure 13a depicts differences of up to 0.6 mm in the North- and East-component as well as up to 0.8 mm for Up-component for three consumer grade GNSS receivers (S/N: 1771, 1779, 2284). The performance of all consumer grade GNSS receivers in this combination limit the coordinate determination. This proves our findings we evaluated previously in the observation domain. Although normal distribution of observation noise is obtained, the combination of geodetic and consumer grade GNSS receivers provide higher noise levels. Hence, the repeatability of daily coordinate solutions are inconsistent. At the same time geodetic GNSS receivers perform very well and provide consistent results (cf. LEIC, JAVA in Figure 13a). These differences are mainly caused by the different amount of available observation per baseline and individual systematic effects at individual GNSS receivers.

Changing only the reference for the processing from SEPT to NEO-M8T (0867) results in Figure 13b. Using a consumer grade GNSS receiver as reference consequently leads to consistent repeatability for all consumer grade GNSS receivers with a level of less than 0.1 mm. Differences between mixed receiver types do not exceed 0.25 mm for the North- and East-component and up to 0.5 mm for the Up-component.

As the mass market receivers have the same manufacture dependent characteristics (e.g., tracking loop parameters, C/N0 thresholds, etc.), only very small and residual and individual differences are noticeable (cf. Figure 13b). The gross of manufacture dependent impacts do cancel out for mass market receivers in this comparison, while for the geodetic receivers, they do not (cf. Figure 13a).

To sum up these findings, we showed that the noise and quality of observables of consumer grade GNSS receivers are not worse than from their geodetic counterparts on both the observation and position domain. Special care has to be applied by mixing up consumer grade and geodetic GNSS receivers. An improved concept for reducing the higher noise is advisable. However, using consistent networks is much more important when using a consumer grade receiver than using a geodetic receiver.

## 5. Discussion of Results

The receiver benchmark analysis provides significant results for the operation and use of geodetic and consumer grade GNSS receivers. The key parameters of the benchmark test are summarized in Table 3.

The set-up of the benchmark test is a temperature stabilised laboratory environment, combined with a characterised GNSS signal splitter and a combination of geodetic and consumer grade GNSS receivers. The analyses were evaluated on the observation and position domain to derive important key parameters within the combination of different kind of GNSS receivers.

The C/N0 as a key parameter of the receiver’s signal tracking performance is a very important tool and measure to adjust the tracking performance parameters correctly, for example, C/N0 threshold and others. Results from the studied C/N0 curves with respect to the code range DD of the consumer grade GNSS receivers indicate that the factory defaults for the minimum C/N0 threshold is not suitable and should be adjusted to fit the requirements of individual approaches. In addition, determining a C/N0 reference curves for receiver and antenna combination provide advantageous parameters for a C/N0 based observation weighting of the observations.

Furthermore, as the consumer grade GNSS receiver provides 32 channels effectively, it is possible to only apply for two different systems when processing the observables in an independent post processor or when the user is interested in a solution independently from the internal receiver solution. Thus, it serves for much more accurate results but with more effort with regard to the data processing chain.

The carrier phase observables of consumer grade GNSS receivers show magnitudes of up to 1.3 mm at zenith following a normal distribution. For code range DDs, residuals of up to 0.5 m are detectable and indicate, that the minimum C/N0 threshold of factory settings is not meaningful. However, normal distribution is present. For GLONASS, the residuals show to have an individual offset although frequency division multiple access techniques are considered in the data processing. Nevertheless, the noise on the carrier phases are determined to 2.0 mm at zenith, and comparable magnitudes with respect to GPS are achieved for the GLONASS code range residuals.

Allan standard deviation and quantile-to-quantile plots prove the hypothesis that the observations follow a normal distributed density function of μ and with standard deviation σ and that white noise is present. All consecutive days of the benchmark analysis show repeatable results with a high temporal stability.

The results are convincing and prove that consumer grade GNSS receivers do not perform significantly worse than their geodetic counterparts. However, there are limitations that require a special consideration: the consumer grade receivers have a smaller number of available channels for tracking satellites. Yet the receivers have a very low C/N0 limit that allows the receivers to track and acquire very weak signals. Typically, there is also no on-board memory to store the data directly on the receiver. Simple adaptations allow a modification of the receiver to set-up an individual extended receiver board design. The presented studies prove a significant higher noise relative to geodetic receivers. However, millisecond deviations of the internal receiver clock time scale are present and need to be corrected in addition. The noise on combined geodetic and consumer grade receivers is significant and should be considered correctly. This leads to the conclusion that for the kind of consumer grade GNSS receivers, consistent networks of manufactures are even more important to achieve precise results than for their geodetic counterparts.

## 6. Conclusions

To conclude our contribution, we found that a comprehensive receiver benchmark analysis serves various and important key parameters for the design of cost-efficient GNSS applications. Results of the determined study figure out that the combination of geodetic and consumer grade GNSS receivers requires some additional effort and caution to receive the desired accuracy and precision in a mixed set-up of consumer grade and geodetic GNSS receivers. Therefore, we summarize the most important findings as follows:

- Consistency of results obtained with consumer GNSS receivers mainly depends on the consistency of the assembled equipment.
- To improve the accuracy and precision of obtained results, it is important to use the same manufacture type.
- For a combination of the same types of consumer grade receivers, carrier phase noise of 2.2 mm (RMS: 1.3 mm) and 0.5–0.75 m (RMS: 0.46 m) code noise is expectable.
- For a combination of consumer and geodetic grade receivers, carrier phase noise of 2.2 mm (RMS: 1.4 mm) and 1.0–1.5 m (RMS: 1.26 m) code noise has to be expected.

In addition, the breaking point between using geodetic high-end or consumer grade and cost-efficient equipment has to be critically evaluated and considered with special care during a rigorous system design. From this study and our experiences, the limiting factor is the higher code noise of the combination between consumer and geodetic grade receivers. However, the carrier phase noise is quite comparable.

The approach of our benchmark set-up figures out that consumer grade receivers of the kind of ublox M8T are an adequate tool within the combination of cost-effective system design, especially when using very short baselines and carrier phase observations. The advantages of the consumer grade receivers are obvious with regard to the use within relative GNSS approaches. Special attention has to be paid when linking the methods to absolute techniques. These results are loosely transferable to other types of consumer grade receivers. However, the paper presents a concept regarding what sorts of system parameters should be analysed and what the benchmark set-up for a verifiable analysis of similar receivers should look like in order to achieve robust results.

Closing the circle from the beginning of this contribution, we conclude that the cost-effective consumer grade receiver approach provides an optimal solution within the system design for direct geo-referencing of 3D point clouds captured with a tls-based MSS.

## Figures and Tables

**Table 1:** Geodetic and consumer market receiver used in in a zero baseline set-up for benchmark study.

Receiver	Type	Frequency	Channels	C/N0 Threshold [dB-Hz]	Firmware Version
UBX	ublox NEO-M8T	single	32	5	3.01/TIM1.01
JAVA	Javad Delta TRE_G3T	multiple	216	25	3.6.11/May 31, 2017
LEIC	Leica GRX1200+GNSS	multiple	120	30	9.20/6.404
SEPT	Septentrio PolaRx5TR	multiple	544	20	5.1.2

**Table 2:** C/N0 deviations in [*dB-Hz*] over elevation bins of 5${}^{{}^{\circ}}$ for a 24 hour data set (DOY 067, 2018). The asterisks for the receiver UBX identifying all the analysed individual receivers.

Receive	Elevation				
	@10${}^{{}^{\circ}}$ [*dB-Hz*]	@15${}^{{}^{\circ}}$ [*dB-Hz*]	@45${}^{{}^{\circ}}$ [*dB-Hz*]	@75${}^{{}^{\circ}}$ [*dB-Hz*]	@90${}^{{}^{\circ}}$ [*dB-Hz*]
SEPT	4	5	2	3	3
JAVA	4	5	2	3	1
LEIC	3	5	2	3	1
UBX 1-5 (****)	5	6	4	6	3

**Table 3:** Summarized key parameters obtained by the benchmark set-up.

Receiver Group A /	Geodetic Grade /	Consumer Grade /	Geodetic Grade /
Receiver Group B	Consumer Grade	Consumer Grade	Geodetic Grade
Carrier phase noise [mm]	2.2	2.2	1.8
Code noise [m]	1.5–2.0	0.5–0.75	0.5–0.75
ΔCMC [m]	±1.0–1.5	±0.5	±0.5
RMS carrier phase noise [mm]	1.4	1.3	1.3
RMS code noise [m]	1.26	0.46	0.34
RMS position [mm]	0.25–0.75	0.1	0.1–0.2

## References

[1] Paffenholz J.A. (2012). Direct Geo-referencing of 3D Point Clouds with 3D Positioning Sensors. Ph.D. Thesis.

[2] Benoit L., Briole P., Martin O., Thom C. (2014). Real-time deformation monitoring by a wireless network of low-cost GPS. J. Appl. Geod..

[3] Schwieger V. Accurate High-Sensitivity GPS for Short Baselines. Proceedings of the FIG Working Week Conference.

[4] Weston N.D., Schwieger V. (2010). Cost Effective GNSS Positioning Techniques. Technical Report, International Federation of Surveyors (FIG), Commission 5 Publication. https://mycoordinates.org/cost-effective-gnss-positioning-techniques/.

[5] Håkansson M. (2018). Characterization of GNSS observations from a Nexus 9 Android tablet. GPS Solut..

[6] Hohensinn R., Geiger A. (2018). Stand-Alone GNSS Sensors as Velocity Seismometers: Real-Time Monitoring and Earthquake Detection. Sensors.

[7] Paziewski J., Sieradzki R., Baryla R. (2019). Signal characterization and assessment of code GNSS positioning with low-power consumption smartphones. GPS Solut..

[8] Cina A., Piras M. (2014). Performance of low-cost GNSS receiver for landslides monitoring: Test and results. Geomat. Nat. Hazards Risk.

[9] Biagi L., Grec F., Negretti M. (2016). Low-Cost GNSS Receivers for Local Monitoring: Experimental Simulation, and Analysis of Displacements. Sensors.

[10] Poluzzi L., Tavasci L., Corsini F., Barbarella M., Gandolfi S. (2019). Low-cost GNSS sensors for monitoring applications. Appl. Geomat..

[11] Krietemeyer A., claire ten Veldhuis M., van der Marel H., Realini E., van de Giesen N. (2018). Potential of Cost-Efficient Single Frequency GNSS Receivers for Water Vapor Monitoring. Remote. Sens..

[12] Douša J., Vàclavovic P., Zhao L., Bezděka P. GOP real-time analyses and data quality check for Europe. Proceedings of the EUREF Symposium.

[13] Wilkinson M.W., McCaffrey K.J.W., Jones R.R., Roberts G.P., Holdsworth R.E., Gregory L.C., Walters R.J., Wedmore L., Goodall H., Iezzi F. (2017). Near-field fault slip of the 2016 Vettore Mw 6.6 earthquake (Central Italy) measured using low-cost GNSS. Sci. Rep..

[14] Schröder D. The new DMT SAFEGUARD low-cost GNSS measuring system and its application in the field of geotechnical deformation and movement monitoring. Proceedings of the 19th EGU General Assembly.

[15] Stempfhuber W., Alberding J. (2012). Geodätische Monitoringsysteme mit RTK Low-Cost-GNSS. Allg. Vermess. Avn.

[16] Kunysz W. Effect of Antenna Performance on the GPS Signal Accuracy. Proceedings of the 1998 National Technical Meeting of The Institute of Navigation (NTM).

[17] Kersten T. (2014). Bestimmung von Codephasen-Variationen bei GNSS-Empfangsantennen und deren Einfluss auf die Positionierung, Navigation und Zeitübertragung. Ph.D. Thesis.

[18] Bochkati M., Schön S. Steering a GNSS Low-cost Receiver with a Chip Scale Atomic Clock and its Impact on PVT Estimation. Proceedings of the Navitec 2018 Signal Workshop.

[19] IGS RINEX—The Receiver Independent Exchange Format Version 3.03. https://kb.igs.org/hc/article_attachments/202583897/RINEX%20303.pdf.

[20] Takasu T. RTKLib: Open Source Program Package for RTK-GPS. Proceedings of the Free and Open Source Software for Geospacial (FOSS4G).

[21] Nischan T. (2019). GFZRNX—RINEX GNSS Data Conversion and Manipulation Toolbox (Version 1.11). Gfz Data Serv..

[22] Kersten T., Paffenholz J. (2018). GNSS Mass Market and Geodetic Receiver Benchmark Study.

[23] Rao B.R., Kunysz W., Fante R., McDonald K. (2013). GPS/GNSS Antennas.

[24] Kaplan E., Hegarty C. (1996). Understanding GPS, Principles and Applications.

[25] Wieser A., Brunner F.K. (2000). An extended weight model for GPS phase observations. Earth Planets Space.

[26] Kersten T., Schön S. GNSS Monitoring of Surface Displacements in Urban Environments. Proceedings of the 18th Internationalen Ingenieurvermessungskurs.

[27] Axelrad P., Larson K.M., Petovello M., Lachapelle G., Murfin T. (2006). GNSS Solution: Orbital precision, optimal dual-frequency techniques, and Galileo receivers. Inside GNSS.

[28] Jakowski N., Teunissen P.J., Montenbruck O. (2017). Ionosphere Monitoring.

[29] Allan D.W. (1966). Statistics of Atomic Frequency Standards. Proc. IEEE.

[30] Allan D.W. (1987). Time and Frequency (Time-Domain) Characterization, Estimation and Prediction of Precision Clocks and Oscillators. IEEE Trans. Ultrason. Ferroelectr. Freq. Control.

[31] Lindsey W., Chie C.M. (1976). Theory of oscillator instability based upon structure functions. Proc. IEEE.

[32] Kersten T., Paffenholz J.A. (2016). Noise Analysis of High Sensitivity GNSS-Receivers for Direct Geo-Referencing of Multi-Sensor Systems. https://www.researchgate.net/publication/307884291_Noise_Analysis_of_High_Sensitivity_GNSS-Receivers_for_Direct_Geo-Referencing_of_Multi-Sensor_Systems?channel=doi&linkId=57d053b308ae0c0081dea804&showFulltext=true.

[33] Dach R., Lutz S., Walser P., Fridez P. (2015). Bernese GNSS Software Version 5.2.

[34] Schaer S., Dach R., Sidorov D., Susnik A., Arnold D., Prange L., Jäggi A., Villiger A. (2017). CODE Final Product Series for the IGS. https://boris.unibe.ch/96351/.

